# Case control feasibility study assessing the association between severity of coronary artery disease with Glutathione Peroxidase-1 (GPX-1) and GPX-1 polymorphism (*Pro198Leu*)

**DOI:** 10.1186/s12872-016-0280-9

**Published:** 2016-05-26

**Authors:** Dinushka Wickremasinghe, Hemantha Peiris, Lal Gotabhaya Chandrasena, Vajira Senaratne, Rasika Perera

**Affiliations:** Faculty of Medical Sciences, University of Sri Jayewardenepura, Nugegoda, Sri Lanka

**Keywords:** Coronary artery disease, Vessel score, Stenosis score, Extent score, Glutathione Peroxidase, GPX-1 *Pro198Leu* polymorphism

## Abstract

**Background:**

Glutathione peroxidase-1 (GPX-1) activity was reported to be useful marker for monitoring cardiovascular disease. However, accurate assessment of coronary artery disease (CAD) using GPX-1 polymorphism is limited for South Asian population. Present study aim to assess GPX-1activity and GPX-1 polymorphismin patients with coronary artery disease (CAD) who were confirmed with coronary angiography findings and in apparently healthy subjects.

**Methods:**

Case control study was carried out with 85 patients (58 males and 27 females) 40–60 years of age confirmed as having CAD on coronary angiography findings and 85 age and sex matched healthy volunteers as controls. Blood samples were analyzed for erythrocyte GPX-1 activity and GPX-1 polymorphism in both groups and the severity of CAD was assessed using coronary angiography scoring system based on vessel, stenosis and extent score.

**Results:**

Coronary angiography scores indicated that erythrocyteGPX-1 cutoff value of 23.9 U/gHb showed a high sensitivity and negative predictive value in ruling out major vessel disease. The GPX-1 *Pro198Leu* (CT) polymorphism was higher in patients with CAD (25.3 %) when compared to controls (10.7 %). *Pro198Leu* (CT) genotype showed a 2.84 fold risk for CAD [odds ratio 2.84 (95 % CI 1.15–6.98), *p* = 0.019].

**Conclusion:**

Coronary angiography findings indicated that individuals possessing *Pro198Leu* (CT) polymorphism were found to be associated with low erythrocyte GPX-1 activity and increased susceptibility for CAD.

## Background

Coronary artery disease (CAD) is a chronic clinical syndrome that could result from interaction of many risk factors. Among them, the conditional risk factors including homocysteine, lipoproteins and inflammatory markers are linked with increased risk for CAD, however the mechanisms which underline the causative and independent contribution to CAD have not been determined [1]. In addition to those conditional risk markers, GPX-1 activity was suggested to be a valuable marker of monitoring cardiovascular events [2].

Meta-analyses studies assessing the activity of GPX-1 in biological fluids and clinical outcomes show that there were substantial between study heterogeneity due to methodological and ethnicity variability [3]. Hence, accurate assessments of these CAD risk markers are needed in combating this chronic clinical syndrome.

GPX-1, the ubiquitous intracellular enzyme is a key antioxidant enzyme present within most cells, including endothelium. Thus, deficiency could enhance atherogenesis [4, 5]. Given that the accumulated evidence based on published research reported on the association between GPX-1 activity and the cardiovascular disease, to our knowledge these studies have only concentrated on the degree of coronary atherosclerosis using conventional angiography findings than the severity of atheroma quantified in best reflection of the atherosclerosis process. Thus these studies did not address the crucial question of whether the erythrocyte GPX-1 activity predicts the severity of CAD.

Evidence suggests that CAD clinical syndrome also results from interaction of many risk factors including genetic factors. In search for genetic factors, the GPX-1 polymorphism was reported to be associated with GPX-1 activity and etiology of many diseases [6]. However, these studies have shown inconsistent results in relation to severity of CAD among East Asians and non-East Asian populations [6]. Thus, identification of GPX-1 genetic risk factors needs urgent priorities for CAD risk stratification.

GPX-1 consists of 38 polymorphisms. However, most of them are not found within the open reading frame in the 5′ and 3′ flanking regions. The most well characterized type is C > T alteration in codon 198, which results in a proline to leucine (*Pro198Leu*) alteration in the polypeptide chain. However, there is no completely elucidated functional consequence of *Pro198Leu*; even though it has been implicated as a risk factor in diseases including CAD [6–8]. Therefore, this study was performed to determine the association of GPX-1activity and GPX-1 *Pro198Leu*polymorphisms with the severity of CAD using coronary angiographic score systems (vessel, stenosis and extent scores) which reflects the proportion of the coronary surface area affected by atheroma.

## Methods

### Subjects

A case control study was carried out with 85 patients (58 males and 27 females) aged 40–60 years who were confirmed as having CAD by coronary angiography findings at Cardiology Unit, National Hospital and Nawaloka Hospitals PLC, Colombo, Sri Lanka during 2013 and 2014.

Subjects with history of cardiovascular, renal, hepatic disease, malignancy, diabetic mellitus, hypertension, dyslipidemia, smoking and alcoholics were excluded from the study. A total of 85 age and sex matched healthy volunteers who had normal exercise ECG and estimated Glomerular Filtration Rate (eGFR) more than 60 ml/min/1.73 m^2^ attending a routine health screening program at Family Health Care Centre, University of Sri Jayewardenepura, Nugegoda, Sri Lanka were recruited as controls to compare the GPX parameters with patients. The sample size of the study was calculated for a matched case control study with a power of 80 %; ratio of cases to controls 1:1; exposure in controls 30 %; expected odds ratio of 2.6 and an alpha error of 5 %.

### Collection of samples and biochemical investigations

Venous blood samples were drawn after an overnight fast (8–10 h) from both patients and controls. Blood samples were immediately divided into two halves and one half was transferred into heparin coated tubes for erythrocyte GPX-1 assay and the remaining was transferred into potassium EDTA tubes for genetic analysis.

### Biochemical assays

#### Glutathione peroxidase-1

The heparinized blood was immediately centrifuged at 3000 g for 5 min and the haemolysate prepared from pre-washed erythrocytes with ice-cold isotonic NaCl for GPX −1 assay. GPX-1 activity was measuredby ELISA method (Northwest Life Sciences Specialties (NWLSS) LLC, Vancouver, USA) using Bio-Rad 680 microplate reader at 450 nm.

#### DNA isolation and Polymerase Chain Reaction (PCR)

Genomic DNA was isolated from peripheral blood leukocytes by using Promega Wizard® Genomic DNA Purification Kit (Promega Corporation, USA) according to manufacturer protocol.

All primers used were purchased from Integrated DNA Technologies, USA. Target fragments were amplified using polymerase chain reaction (PCR). Two primers, forward 5′-AGCCCAACTTCATGCTCTTC-3′ and reverse 5′- CAGGTGTTCCTCCCTCGTAG- 3′ were used to amplify the 400-base pair (bp) fragment containing the C/T polymorphic site.

The PCR reaction was performed in a 25 μL of reaction mixture containing; 2.5 μl of 10 X PCR buffer (500 mM KCl, 100 mM Tris–HCl, 1.0 % Triton X-100; Promega), 2.5 mM MgCl2, 2.5 μl 3.5 mM dNTPs, 2.5 μl 5 μM of each primer, 0.4 μl of 2 U of Taq polymerase in storage buffer B [20 mM Tris–HCl, 100 mM KCl, 0.1 m Methylenediaminetetraacetic acid, 1 mM dithiothreitol (DTT), 50 % glycerol, 0.5 % Nonidet-P40, and 0.5 % Tween 20; Promega] and 3 μl of isolated DNA. Finally, 14.1 μl of sterile H_2_O was added to make 25 μL of total volume of PCR mixture.

**Fig. 1:**
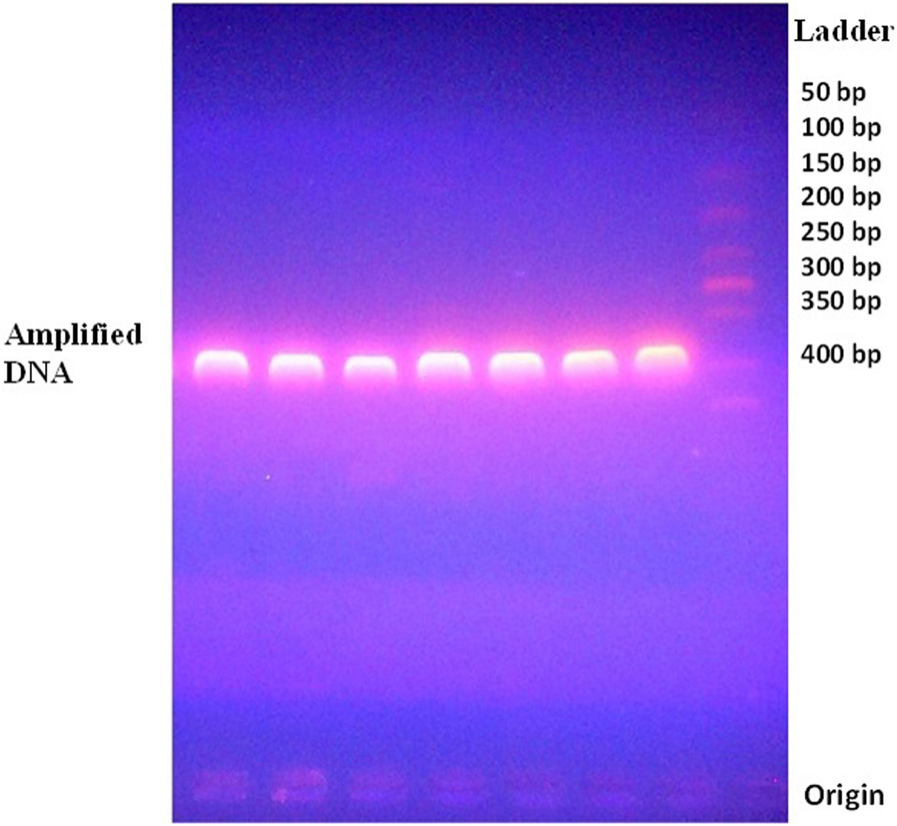
Amplified DNA by PCR. Extracted DNA amplified by PCR and resolved in 2 % agarose gel stained with ethidium bromide at 50 V for 2 h. Gel electrophoresis given 400 bp bands as target sequence

PCR was carried out in Applied Biosystem verity® thermo cycler (USA). The PCR cycling conditions of the assay were 94 °C for 5 min, followed by 35 cycles at 94 °C for 30 s, 55 °C for 30 s, and 72 °C for 45 s, with a final extension step at 72 °C for 7 min. Amplicons of size 400 bp was verified by running 5 μl of PCR product on a 2 % agarose gel (Fig. 1).

#### Restriction fragment length polymorphism (RFLP)

**Fig. 2:**
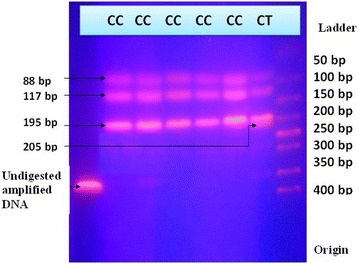
Agarose gel electrophoresis image of restriction fragment length polymorphism. Amplified DNA by PCR was digested with the restriction enzyme 12 U of *Apa*I overnight at 37 °C and resolved in 2 % agarose gel stained with ethidium bromide at 50 V for 2 h. Resolved band patterns were photographed under ultra violet light by the Elite camera systems

The amplified PCR product was digested with the restriction enzyme 12 U of ApaI (Promega Corporation, Madison, USA) overnight at 37 °C and resolved for 2 h at 50 V on 2 % agarose gel stained with ethidium bromide. Restriction fragments were visualized under UV light and photographs made (Elite camera systems). The created patterns of bands were interpreted to identify respective genotypes (Fig. 2).

Analysis of the number of fragments present as well as the size of these fragments allowed for genotype determination. The *Pro198Pro* (CC) genotype yielded by 195, 117, and 88-bp fragments; the *Pro198Leu* (CT) genotype yielded by 205, 195, 117, and 88-bp fragments; although no any subject belongs to the *Leu198Leu* (TT) genotype with expected band pattern 205 and 195-bp.

The procedures followed were in accordance with the ethical standards of the declaration of Helsinki of the World Medical Association.

### Assessment of severity of coronary artery disease

Coronary angiography reports and the compact disc recordings of angiograms were independently reviewed by two interventional cardiologist, who had no access to the patients’ clinical and laboratory findings. The angiograms were scored as described below.

#### Vessel score

Vessels with a significant stenosis (70 % or greater reduction in lumen diameter) was given a score of 1. Four coronary arteries were considered in this system; if significant stenosis in any of three arteries [Left Anterior Descending (LADA) Artery or Left Circumflex Artery (LCA) or Right Coronary Artery) given score 1 for each occasion, while significant stenosis in main left coronary artery was considered as single vessel disease (score 1) and disregard others (LADA and LAC). Thus, Vessel score range from 0 to 3; as 0 for no vessel disease, 1 for single vessel disease, 2 for double vessel disease and 3 for triple vessel disease.

#### Stenosis score

The stenosis score was calculated by a modified Gensini score as described by Reardon et al. 1985 [9] and Hamsten et al. 1986 [10], “which places emphasis on the severity of stenosis while including some measure of the extent of coronary artery disease. Briefly, the most severe stenosis in each eight coronary segments was scored according to;score 1 for 1–49 % reduction in luminal diameter, 2 for 50–74 %, 3 for 75–99 % and 4 for total occlusion. The scores for each of the eight segments were added together to give a total score out of a theoretical maximum of 32”.

#### Extent score

The extent score was calculated according to the method described by Sullivan et al. 1990 [11] which indicates the “proportion of the coronary artery tree involved by angiographycally detectable atheroma. The proportion of each vessel involved by atheroma, identified as luminal irregularity was multiplied by a factor for each vessel: left main artery, 5; left anterior descending artery, 20; main diagonal branch, 10; first septal perforate,5; left circumflex artery, 20; obtuse marginal and posterolateral vessels,10; right coronary artery, 20; and main posterior descending branch,10. When the major lateral wall branch was a large obtuse marginal on intermediate vessels, this was given a factor of 20 and the left circumflex artery a factor of 10. When a vessel was occluded and the distal vessel not fully visualized by collateral flow, the proportion of vessel not visualized was given the mean extent score of the remaining vessels. The scores for each vessel or branch were added to give a total score out of 100, being the percentage of the coronary intimal surface area involved by atheroma”.

### Data processing and statistical analysis

Reference intervals for GPX-1 levels in control subjects were determined using 95 % confidence intervals (CI’s). Multivariate logistic regression analysis was performed to eliminate the influence of confounding factors for CAD. Continuous variables were analyzed using independent sample t test, Analysis of variance (ANOVA) and Pearson’s correlations. Fisher’s exact tests were performed to compare the distribution of GPX-1 genotypes and allele frequencies. A *p* value ≤ 0.05 was considered to be significant.

Conventional coronary angiography has been considered as the gold standard for diagnosis of CAD [12]. The accuracy of detecting the severity of CAD in patients using GPX-1activity was determined by measuring the area under the Receiver Operating Characteristics (ROC) curve, 95 % confidence interval, sensitivity, specificity and positive and negative predictive values and likelihood ratio based on the coronary angiography findings.

### Ethics consent and permissions

This study protocol was approved by the Ethics Review Committee of the Faculty of Medical Sciences, University of Sri Jayewardenepura, Sri Lanka. Procedures followed were in accordance with the Ethics standards on human experimentation and conforms to the guidelines of the declaration of Helsinki [13]. All participants were informed about the study and written consent obtained.

## Results

### Characteristic of subjects

This study was carried out in 85 patients with CAD (58 males and 27 females) and 85 age and sex matched healthy individuals as controls; the age range of the study population was 40–60 years (mean age 53 ± 2 years). Among the risk factors assessed, patients with a family history of premature heart disease showed a significant association with CAD when compared to controls [OR 5.15 (95 % CI 2.0–12.8), *p* = 0.001] (Table 1). Multivariate logistic regression analysis, after eliminating the influences of confounding factors for CAD shows that the positive family history of CAD and erythrocyte GPX-1 activity and GPX *Pro198Leu* (CT) genotype had significantly high Odds ratio (Table 2) indicating that GPX-1 and GPX *Pro198Leu* (CT) genotype appear to important markers of CAD (Table 2).

**Table 1:** Demographic features and distribution of CAD risk factors in patients and controls

Variable	Patients		Controls		Odds ratio	*p* value
	*n*	%	*n*	%	(95 % CI)	
Gender						
Male	58	68	58	68	1.00	1.000
Female	27	32	27	32	(0.4–2.1)	
Family history of heart disease						
Yes	30	34.7	8	9.3	5.15	0.001
No	55	65.3	77	90.7	(2.0–12.8)	
Family history of diabetes mellitus						
Yes	25	29.3	18	21.3	1.53	0.262
No	60	70.7	67	78.7	(0.72–3.21)	
Veganism						
Yes	8	9.3	7	8.0	0.845	0.772
No	77	90.7	78	92.0	(0.27–2.64)	

Pearson chi square test comparing cases and controls.^*^Significant at *p* ≤ 0.05

**Table 2:** Associations of risk models for coronary artery disease in study population by multivariate logistic regression analysis

Variable	Odds ratio	95 % confidence interval	*p* value
Age (Years)^a^	0.97	0.92–1.02	0.322
Gender (Male)	0.77	0.25–2.40	0.652
Body mass index (kg/m^2^)^a^	0.99	0.90–1.09	0.893
Positive Family history of CAD	7.20	2.07–25.10	0.002
GPX-1 < 23.9 U/g Hb	4.72	1.61–13.79	0.005
Pro198Leu (CT) genotype	2.14	0.60–7.55	0.035

^a^Consider as continuous variable. Significant at *p* ≤ 0.05

### Assessment of the severity of coronary artery disease based on scoring system

Table 3, Shows the summary statistics of three severity scores (vessel, stenosis, extent) of CAD patients.

**Table 3:** Severity Scores of Coronary artery disease patients

	Mean	±SD	Ranges			95 % Confidence Interval
			Minimum		Maximum	
Vessel score	1.72	0.78	1	-	3	1.50–1.88
Stenosis score	8.43	4.76	3	–	24	7.33–9.52
Extent score	52.13	18.56	20	–	95	47.86–56.41

Severity of CAD was assessed by vessel, stenosis and extent score. Each score was divided into three categories as shown in Table 4. When vessel score is considered, 52 % had double or triple vessel disease. As far as stenosis score is concerned, 37 % were diagnosed as having moderate to severe disease. Extent score shows that the vast majority (94 %) had moderate to severe disease. Thus, it can be assumed that severity of CAD among these patients is higher.

**Table 4:**
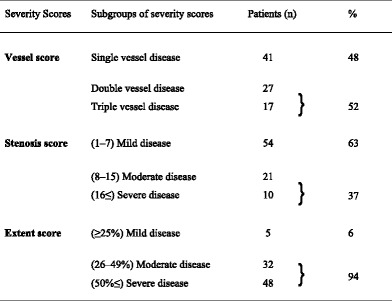
Frequency distributions of the three severityscore systems in patients with CAD

### Interpretation of ROC Curves with respect to concentrations of erythrocyteGPX-1 activity based on vessel, stenosis and extent score

The diagnostic accuracy of GPX-1 for CAD based was determined by measuring the area under the ROC curve (AUC), 95 % confidence interval, sensitivity (SE%), specificity (SP%), positive predictive value (PPV%), negative predictive values (NPV%), Positive likelihood ratio (PLR) and negative likelihood ratio (NLR) (Table 5).

**Table 5:** Receiver operating characteristic curves generated optimum cut-off values for coronary artery disease risk markers with severity of CAD scoring systems

Cut-off values	(%) SE 95 % CI	(%) SP 95 % CI	(%) PPV 95 % CI	(%) NPV 95 % CI	PLR 95 % CI	NLR 95 % CI	AUC 95 % CI	*P* value
Vessel score								
GPX-1	97	61	73	96	2.5	0.04	0.931	0.000
23.9 (U/g Hb)	86–99	43–77	59–84	78–99	1.7–3.8	0.0–0.3	0.867–0.995	
Stenosis score								
GPX-1	91	42	42	91	1.6	0.20	0.768	0.000
23.9 (U/g Hb)	73–99	28–56	29–57	72–99	1.2–2.0	0.0–0.7	0.651–0.885	
Extent score								
GPX-1	78	43	63	61	1.4	0.5	0.681	0.007
23.9 (U/g Hb)	63–90	25–61	49–76	38–80	1.0–1.9	0.2–1.0	0.558–0.805	

*AUC* Area under curve, *95 % CI* 95 % confidence interval, *NLR* Negative likelihood ratio, *NPV* Negative predictive value, *PLR* Positive likelihood ratio, *PPV* Positive predictive value, *SE* Sensitivity, *SP* Specificity; Significant at *p* < 0.05

According to ROC given in vessel score the AUC for GPX-1 was 0.93 with a detectable cutoff value of 23.9 U/gHb keeping acceptable sensitivity (97 %), PPV and NPV of 74 % and 96 % respectively suggesting that GPX-1 appeared to be an important predictive marker of ruling out CAD in present study population (Table 5).

When considering the stenosis score the GPX-1 cutoff value of 23.9 U/gHb showed a high sensitivity and negative predictive value (SE 91 %, NPV 91.3 %, NLR = 0.20, AUC = 0.768) suggesting that the GPX-1has a value in ruling out major vessel disease and luminal narrowing by atheroma (Table 5).

However, GPX-1 shown a moderate but significant sensitivity and NPV (SE 78 %, NPV 60.8 %, NLR = 0.5; AUC = 0.681) with a moderate accuracy for predicting severity of CAD for extent score compared to stenosis score. Thus, when considering severity of CAD, erythrocyte GPX-1cut-off value of 23.9 U/gHbis an important predictor in ruling out major vessel disease and luminal narrowing by atheroma.

### Association of allelic frequency of GPX-1 Pro198Leu polymorphism and severity of CAD

Summary of genotyping results is presented in Table 6. The allelic frequencyof *Pro198Leu* polymorphism shows that the CC genotype was the most frequent, followed by CT, although TT genotype was not isolated from the study population. Frequency distribution of *Pro198Pro* (CC) genotype was significantly higher in controls (89.3 %) when compared to patients with CAD (74.7 %). However, *Pro198Leu* (CT) genotype was significantly low in controls (10.7 %) when compared with patients (25.3 %) (χ^2^test value = 1.019). Interestingly, *Pro198Leu* (CT) genotype showed a 2.84 fold risk for CAD [odds ratio 2.84 (95 % CI 1.15–6.98), *p* = 0.019] (Table 6). When allelic frequency in CAD patients and controls were analyzed the common Pro198 (C) encoding variant was higher in controls (94.6 %) when compared to patients with CAD (87.3 %) and, on other hand, Leu198 (T), encoding variant was 12.7 % and 5.4 % respectively in patients with CAD and controls.

**Table 6:** Frequency distribution of Glutathione peroxidase 1 variants in study subjects

	Patients		Controls		Odds ratio	(95 % CI)	*p* value
	No	%	No	%			
Pro198Pro	63	74.7	76	89.3	2.84	1.15–6.98	0.019
(CC)							
Pro198Leu	22	25.3	9	10.7			
(CT)							
Leu198Leu	0	0	0	0			
(TT)							

Pearson’s chi square test. Significant at *p* ≤ 0.05

CAD severity scores were compared between *Pro198Pro* (CC) genotype and *Pro198Leu* (CT) genotype in CAD patients. CAD severity was significantly high in *Pro198Leu* (CT) genotype patients compared to *Pro198Pro* (CC) genotype patients assessing with stenosis score (*p* = 0.018). While, assessing for vessel score and extent score there were insignificant differences with trend to high severity for *Pro198Leu* (CT) genotype patients (Table 7).

**Table 7:** Association of GPX-1 Pro198leu polymorphism with severity of coronary artery disease

	Mean severity score	±SD	*p* value
Vessel score			
Pro198Pro (CC)	1.47	0.69	0.112
Pro198Leu (CT)	1.80	0.79	
Stenosis score			
Pro198Pro (CC)	6.21	2.3	0.018
Pro198Leu (CT)	9.18	5.1	
Extent score			
Pro198Pro (CC)	46.32	15.6	0.115
Pro198Leu (CT)	54.10	19.1	

Independent sample t test. Significant at *p* ≤ 0.05

In addition erythrocyte GPX-1 activity in patients also shown a significant reduction with increased severity of CAD assessed in all three scoring systems viz, vessel, the stenosis and the extent score indicating that GPX-1 appeared to be a more sensitive marker of CAD (Table 8). In both the patients with CAD and control groups, there was no significant deviation of GPX-1 genotype frequencies from those predicted by the Hardy-Weinberg equilibrium (Fisher’s exact test; *p* = 0.720 for CAD patients and *p* = 1.000 for controls, respectively).

**Table 8:** GPX-1 activities in subgroups of three score systems of severity of CAD

Vessel score subgroups				
	SVD	DVD	TVD	*p* value
GPX-1 (U/g Hb)	28.4 ± 9.5	15.0 ± 4.4	13.1 ± 3.4	0.000
Stenosis score subgroups				
	(1–7) Mild	(8–15) Moderate	(16≤) Severe	*p* value
GPX-1 (U/g Hb)	23.5 ± 10.4	17.5 ± 10.5	10.9 ± 2.9	0.006
Extent score subgroups				
	(≤25 %) Mild	(26–49 %) Moderate	(50 %≤) Severe	*p* value
GPX-1 (U/g Hb)	22.4 ± 9.7	16.3 ± 11.1	10.5 ± 9.3	0.020

*DVD* Double Vessel Disease, *SVD* Single Vessel Disease, *TVD* Triple Vessel Disease

One way Analysis of variance (ANOVA) test. Significant at *p* ≤ 0.05 level

## Discussion

In recent years many aspects of the pathogenesis of atherosclerosis have been researched [14]. Among them oxidative stress has been elucidated as one of the potential mechanisms of atherosclerosis. Although evidence of previous studies have proved that a positive relationship exists between a number of cardiovascular risk markers including GPX-1and the development of CAD, these studies had limitations in assessing the extent of coronary atherosclerosis [15]. Thus, these findings raise questions about the conventional risk assessment in patients. Subsequently, new angiographic scoring methodwas used to assessing the extent of coronary atherosclerosis [16]. However, the vessel scores as described before in other studies is a conventional way of assessing the severity of ischemia compared to the stenosis and extent scores which measure the extent of atheroma. Thus, the present study was undertaken toassess the association between severity of coronary artery disease with GPX-1 and GPX-1 polymorphism (*Pro198Leu*) based on new angiography scoring system [9–11]. To the best of our knowledge this is the most recent study has performed to assess association of severity of CAD using erythrocyte GPX-1 activity and GPX-1 polymorphism in a study based onvessel, stenosis and extent score based onproportional of the coronary artery tree involved by angiographycally detectable atheroma.

In present study GPX-1 activity showed a significantly inverse relationship (*P* < 0.000) with the severity of CAD. Evidence also suggests that elevated ROS act synergistically with the standard risk factors of CAD [17, 18]. Previous studies reported that erythrocyte GPX-1 activity was lower in patients with multi vascular atherosclerosis and was inversely correlated with the cardiovascular event rate [4, 16]. Thus, the reduction of erythrocyte GPX-1 activity in patients with triple vessel disease compared to double and single vessel disease indicating low GPX-1 activity could be one of the possible causesof coronary atherosclerosis in our study group.

In humans, GPX-1 gene contains polymorphism of cytosine-to-thymine (C < T) substitution at codon 198, results in Pro198Lue variation. Thus, the Leu variant was reported to be associated with reduction of GPX-1 activity and increased susceptibility to diseases [6, 17, 19]. However, the association of GPX-1 polymorphism and the coronary vascular disease (CVD) risk reported in previous studies found conflicting results [6, 20, 21]. Perhaps, this may be possibleas the frequency distribution of T allele in GPX-1 varied among the East Asians and Non-East Asians [22]. Thus, ethnic differences are considered to be a vital factor to produced heterogeneity. The distribution of Pro, Leu homozygotes and heterozygotes in both patients and controls in our study were in accordance with the Hardy-Weinberg equilibrium. According to results, the*Leu198Leu* (TT) genotype was not identified in our study population which is an important finding for future research.

Stenosis score was significantly high in *Pro198Leu* (CT) genotype patients and both vessel and extent score show insignificant trend to high severity for *Pro198Leu* (CT) genotype patients. Stenosis score calculates the number of coronary arteries with extensive stenosis with broad percentages while vessel score merely calculates the number out of four major coronary arteries involved; yet the extent score represent the coronary intimal surface area affected by atheroma as indicated by the area of supply and percentage of luminal narrowing. Hence, CAD patients carrying *Pro198Leu* (CT) genotype are susceptible to increased severity of CAD.

GPX-1 activity did not show significant differences between two genotypes in both vessel and extent score groups, however the *Pro198Leu* (CT) genotype showed a trend of reduction in GPX-1 activity with increase severity in all three score groups indicating that T allele (Proline to Leucine substitution) was less responsive in stimulation of erythrocyte GPX-1 activity in patients with CAD. Thus, the GPX-1 *Pro198Leu* polymorphism is appeared to be one of the significant causes of severity of CAD.

One of the limitations of the study is its smaller sample size. Yet this study was conducted as a feasibility study with the aim of expanding it to a higher sample depending on the results. Another limitation was the possible influence of physical activity on GPX-1 level as all subjects in the study were physically active individuals. However, its effect may be less as all subjects were after a 10 h overnight fast and avoided vigorous exercise or activities before sample collection.

Serum selenium levels have been reported to be associated with GPX-1 activity in humans [7]. Yet data on the correlation between selenium and GPX-variants among Sri Lankan populations are currently unavailable. Hence, this study may open a new window for further research.

## Conclusion

According to coronary angiography scoring method, the GPX-1 *Pro198Leu* (CT) polymorphism showed a trend in reduction of GPX-1 activity with increase severity of CAD, suggesting that individuals possessing the *Pro198Leu* (CT) genotype, especially in East Asian populationsare appear to be more susceptible for CAD. Thus, we propose lager sample size with East Asian and non-East Asian populationstudy to identify novel polymorphisms located in potential regulatory regions of the GPX-1 gene, which may modify gene expression and their association with CAD risk.

## Abbreviations

AUC, area under curve; CAD, coronary artery disease; CI’s, confidence intervals; CVD, coronary vascular disease; eGFR, estimated glomerular filtration rate; GPX-1, Glutathione peroxidase-1; NLR, negative likelihood ratio; NPV, negative predictive values; PLR, positive likelihood ratio; PPV, positive predictive value; ROC, receiver operating characteristics; SE, sensitivity; SP, specificity.

## References

[1] Grundy SM, Pasternak R, Greenland P, Smith S, Fuster V (1999). Assessment of cardiovascular risk by use of multiple risk factor assessment equation. A statement for healthcare professionals from the American Heart Association and the American College of cardiology. Circulation.

[2] Blankenberg S, Rupprechi MJ, Bickel C, Torzewski M, Hafner G, Tiret L (2003). Glutathione Peroxidase 1 activity and Cardiovascular Events in patients with Coronary Artery Disease. N Engl J Med.

[3] Weinbrenner T, Cladellas M, Isabel CM, Fito M, Tomas M, Senti M (2003). High oxidative stress in patients with stable coronary artery disease. Atherosclerosis.

[4] Chandrasena LG, Peiris H, Waikar HD (2009). Biochemical changes associated with reperfusion after off-pump and on-pump coronary artery bypass graft surgery. Annal Clin lab Sci.

[5] Schnable R, Lachner KJ, Rupprecht HJ, Espinola-Klein C, Torzewski M, Lubos E (2005). Glutathione peroxidase-1 and homocysteine for cardiovascular risk prediction results from the Atherogene study. J Am Coll Cardiol.

[6] Zhang JX, Wang ZM, Zhang JJ, Zhu LL, Gao XF, Chen SL (2014). Association of glutathione peroxidase-1 (GPx-1) rs1050450 Pro181Leu and Pro1971Leu polymorphisms with cardiovascular risk: a meta-analysis of observational studies. J Geriatr Cardiol.

[7] Jablonska E, Gromadzinska J, Reszka E, Wasowicz W, Sobala W, Szeszenia-Dabrowska N (2009). Association between GPx1 Pro198Leu polymorphism, GPx1 activity and plasma selenium concentration in humans. Eur J Nutr.

[8] Foster CB, Aswath K, Chanock SJ, Mckay HF, Peters U (2006). Polymorphism analysis of six selenoprotein genes: support for a selective sweep at the glutathione peroxidase 1 locus (3p21) in Asian populations. BMC Genet.

[9] Reardon MF, Nestel PJ, Craig IH, Harper RW (1985). Lipoprotein predictors of the severity of coronary artery disease in men and women. Circulation.

[10] Hamsten A, Walldius G, Szamosi A, Dahlen G, de Faire U (1986). Relationship of angiographically defined coronary artery disease to serum lipoproteins and apolipoproteins in young survivors of myocardial infarction. Circulation.

[11] Sullivan DR, Marwicks TH, Freedman SB (1990). A new method of scoring coronary angiograms to reflect extent of coronary atherosclerosis and improve correlation with major risk factors. Am Heart J.

[12] Sokhanvar S, Mazaki RRS, Mousavinasb N, Golmohammadi Z (2011). The association between serum lipoprotein (a) and other cardiac risk factors with the severity of coronary artery disease. J Cardiovasc Thorac Res.

[13] World Medical Association (2013). World Medical Association Declaration of Helsinki Ethical Principles for Medical Research Involving Human Subjects. JAMA.

[14] Sivapalaratnam S, Boekholdt SM, Trip MD, Sandhu MS, Luben R, Kastelen JJ (2010). Family history of premature coronary artery disease and risk prediction in the EPIC-Norfolk prospective study. Heart.

[15] Hamanishi T, Furuta H, Kato H, Doi A, Tamai M, Shimomura H (2004). Functional Variants in the Glutathione Peroxidase-1 (GPX-1) Gene Are Associated With Increased Intima-Media Thickness of Carotid Arteries and Risk of Macrovascular Diseases in Japanese Type 2 Diabetic Patients. Diabetes.

[16] Gemma FM, Paloma CS, Robetto E, Eliseo G, Jaume M, Joachim B (2009). Antioxidant enzyme activity and coronary heart disease: meta-analysis of observational studies. Am J Epidemiol.

[17] Ravn HG, Olsen A, Tjonneland A, Dragsted LO, Nexo BA, Wallin H (2006). Associations between GPX1 Pro198Leu polymorphism, erythrocyte GPX activity, alcohol consumption and breast cancer risk in a prospective cohort study. Carcinogenesis.

[18] Madamanchi NR, Vendrov A, Runge MS (2005). Oxidative stress and vascular disease. Arterioscler Thromb Vasc Biol.

[19] Chen J, Cao Q, Qin C, Shao P, Wu Y, Wang M (2011). GPX-1 polymorphism (rs1050450) contributes to tumor susceptibility: evidence from meta-analysis. J Cancer Res Clin Oncol.

[20] Tang NP, Wang LS, Yang L, Gu HJ, Sun QM, Cong RH (2008). Genetic variant in glutathione peroxidasel gene is associated with an increased risk of coronary artery disease I a chines population. Clin Chim Acta.

[21] Yumie T, Irena BK, Lampe JW, Burk RF, Hill KE, Santella RM (2012). Genetic variation in GPX1 is associated with GPX1 activity in a comprehensive analysis of genetic variations in selenoenzyme genes and their activity and oxidative stress in humans. J Nutr.

[22] Ichimura Y, Habuchi T, Tsuchiya N, Wang L, Oyama C, Sato K (2004). Increased risk of bladder cancer associated with a glutathione peroxidase 1 codon 198 variant. J Urol.

